# Dental Implant with Porous Structure and Anchorage: Design and Bench Testing in a Calf Rib Model Study

**DOI:** 10.3390/ma18030700

**Published:** 2025-02-05

**Authors:** Keila Lovera, Vicente Vanaclocha, Carlos M. Atienza, Amparo Vanaclocha, Pablo Jordá-Gómez, Nieves Saiz-Sapena, Leyre Vanaclocha

**Affiliations:** 1 CDL Clínica Dental Lovera, Avenida Cornellà, 2-BJ, Esplugues de Llobregat, 08950 Barcelona, Spain; keilalovera@gmail.com; 2 Faculty of Medicine and Odontology, Department of Surgery, University of Valencia, 46010 Valencia, Spain; 3 Biomechanics Institute of Valencia, Polytechnic University of Valencia, 46022 Valencia, Spain; carlos.atienza@ibv.org (C.M.A.); amparovanaclocha@hotmail.com (A.V.); 4 Hospital General Universitario de Castellón, 12004 Castellón de la Plana, Spain; jorda.gomez.pablo@gmail.com; 5 Hospital General Universitario de Valencia, 46014 Valencia, Spain; nssapena@hotmail.com; 6 Medius Klinik, Ostfildern-Ruit Klinik für Urologie, Hedelfinger Strasse 166, 73760 Ostfildern, Germany; leyrevanaclocha@hotmail.com

**Keywords:** bone–implant interactions, osseointegration, titanium implants, porous implants, 3D printing

## Abstract

Primary dental implant stability is critical to enable osseointegration. We assessed the primary stability of our newly designed dental implant. We used the calf rib bone animal model. Our implant has an outside tapered screw with two inside barrettes that deploy with a second screw situated at the implant’s crown. We used ten calf ribs with III/IV bone density and inserted ten implants per rib. We deployed the barrettes in the calf rib’s transversal direction to support against the nearby cortical bone. We measured the primary implant’s stability with resonance frequency analysis and collected the Implant Stability Quota (ISQ) in the transverse and longitudinal calf rib planes before (PRE) and after (POS) deploying the barrette. The mean ISQ was PRE 84.00 ± 3.56 and POS 84.73 ± 4.53 (*p* = 0.84) in the longitudinal plane and PRE 81.80 ± 2.74 and POS 83.53 ± 4.53 (0.27) in the transverse plane. The barrettes’ insertion increases our dental implant primary stability by 11% in the transverse plane and 2% in the longitudinal plane. Our dental implant ISQ values are in the higher range than those reported in the literature and reflect high primary stability after insertion. The barrette deployment improves the dental implant’s primary stability, particularly in the direction in which it deploys (transverse plane).

## 1. Introduction

Dental implants are an excellent solution to compile tooth decay with the eventual loss of teeth [1,2]. They are commonly needed among older people when osteoporosis is frequent [3,4], particularly in the maxilla [5]. Osteoporosis makes primary dental implant stability paramount [6,7].

Primary dental implant stability depends on press-fit relative to nearby bones with threads [8,9], while the secondary one is dictated mainly by osseointegration with new bone growth [10]. Brånemark [11] described osseointegration as “the intimate, direct, functional connection, maintained over time, between the bone and an implant subjected or not to load”. The implant’s surface roughness and porosity, particularly materials like titanium [12] and tantalum [13], provide an exceptional milieu for osseointegration [14,15]. However, immediate primary stability is essential to allow osseointegration [9,16], which takes several months [10,17].

The primary stability of most dental implants relies on a screw-like design with threads [9,18] and tapered shapes [19,20,21]. It depends mainly on the cortical bone of the mandible or maxilla [22,23,24], as the cancellous one has little resistance [25,26]. Unfortunately, the bones in old osteoporotic patients are weak and provide limited primary implant stability [6,16], which is vital for the long-term success of any dental implant [16]. Osteoporotic bones are inherently weaker and more prone to failure, necessitating advanced surgical techniques, appropriate implant design, and materials to compensate for the lack of adequate bone density [9].

Additionally, micromotion during osseointegration induces fibrous tissue formation instead of real bone [27,28]. This fibrous tissue will not hold the implant, and dental implant failure might follow [29,30].

Autologous or heterologous iliac crest bone augmentation is an alternative for patients with severe mandibular or maxillary bone atrophy [31,32]. Although both provide satisfactory implant survival rates, the heterologous ones are more prone to bone resorption and clinical complications compared to the autologous ones [31]. Increasing dental implant survival in mandibular and maxillary iliac crest bone grafts involves a multi-faceted approach, including optimal graft handling, advanced surgical techniques, and addressing systemic factors [33]. By ensuring proper graft placement, enhancing vascularization, using biomaterials, minimizing infection risks, selecting an appropriate dental implant [9,34], and carefully timing implant placement, clinicians can significantly improve long-term outcomes and implant success rates [35]. Yet, the ideal situation would be to have a dental implant that could be inserted into very osteoporotic bone without failures and with successful osseointegration [36]. This implant would avoid the two operations and the bone harvest needed for iliac crest bone augmentations [37].

To add an extra primary stability mechanism, we designed a new dental implant with a porous surface and an anchoring system consisting of two deployable barrettes. Those barrettes expand in lingual and vestibular directions and rest on the nearby cortical bone. We expect that the tapered shape, the porous body, the width and size of the threads, and the barrettes will improve the primary implant stability, particularly in the transversal direction of the maxilla or mandibula, which is where most dental implants fail in the medium and long terms [38]. To confirm this, we evaluated the primary stability of our dental implant in the animal model of the calf rib bone. We compared our data with the data available in the literature.

## 2. Materials and Methods

**Figure 1:**
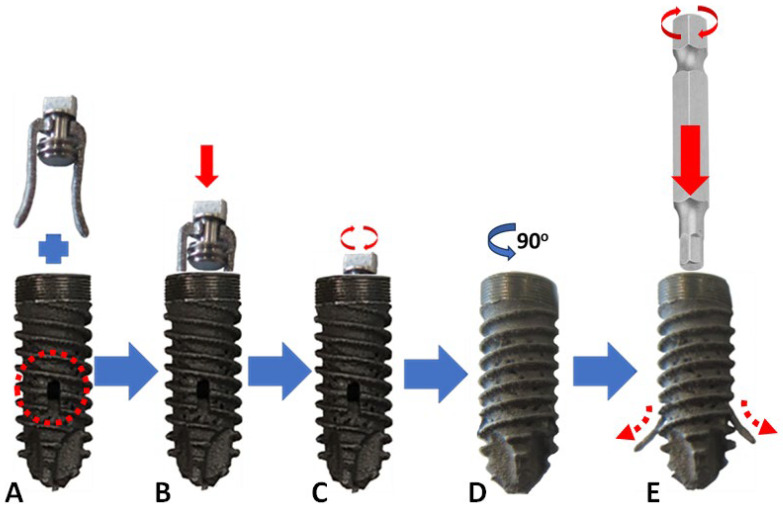
Components of our dental implant: 4.5 mm diameter × 12 mm long screw and two barrettes with their deploying system. Straight red arrows indicate movement in that direction. Curved arrows indicate the direction of rotation of the dental implant, or the screwdriver used to deploy the barrettes. (**A**) The body of the dental implant with barrettes outside it, and the red circle shows the side hole for the barrette to come out. (**B**) Barrettes inserted inside the body of the dental implant. (**C**) Screwing the central screw that secures and deploys the two barrettes. (**D**) The dental implant is rotated 90° along its axis to show how the barrettes come out (shown here for a better understanding of the mechanism; in real life, the implant will not be rotated once in its definitive position). (**E**) The central hexagonal screw head is screwed, and the barrettes are deployed.

Our dental implant has two components (Figure 1), and it was described thoroughly in a previous publication [39]. It is manufactured out of titanium alloy (Ti6Al4V, elasticity module 1100 MPa). It has a dental implant body, barrettes, and a deployment screw. The dental implant body consists of an implant–abutment connection thread in the head of the implant, two side holes (in opposite directions) for the deployment of the two barrettes, a double thread with a 1 mm pitch, and a self-drilling tip thread. It has a porous area to facilitate osseointegration, a 16° angle hexagonal implant–abutment connection with Morse taper fixation, and an internal thread for anchor insertion and attachment connection.

The first is a titanium (Ti6Al4V) screw that is 4.5 mm in diameter and 12 mm in length, with a tapered end and porous surface. The second component is two barrettes inserted inside the screw that deploy into the nearby cancellous bone, screwing a second screw and forcing the barrettes to come out.

We used ten young calf rib specimens with bone density type III/IV [40,41], the bone quality corresponding to our target patients. Calf ribs provide a practical, ethical, and cost-effective model for studying dental implants [40,42]. Their anatomical, biomechanical, and histological similarities to human jawbones make them a suitable model for implant research, allowing for experiments that simulate real-world conditions [43]. This fact justifies their use in implantology, although human cadaveric maxilla or mandible bones are viable [44] but less commonly used due to cost or availability concerns.

The animals from the local abattoir (Plaça Num 106 Res Urb, 3, Quatre Carreres, 46013 Valencia, Spain) were 14 months old, weighing 295 ± 17 kg, and sacrificed for human consumption. This animal model has a proportion of cortical and cancellous bone similar to the edentulous human jaw [45].

Immediately after the sacrifice, we harvested the 60 cm central part of the 8th and 9th ribs, cleaned them of soft tissues, and stored them in closed plastic bags at −18 °C.

**Figure 2:**
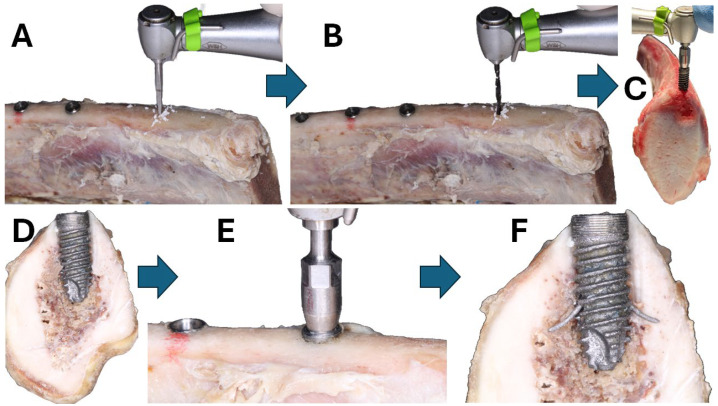
(**A**) Initial drill. (**B**) Second drill. (**C**) Implant insertion. (**D**) Implant inserted in the calf rib. (**E**) Deployment of the barrettes screwing the central screw. (**F**) Final implant position after barrettes’ deployment.

The same person performed all surgical maneuvers (KL). First, we thawed the stored ribs at room temperature and 60% humidity for 4 h. Then, we secured each rib between metallic clamps under gentle pressure and used the rib sections that were not pressed by the clamps to prevent false results. During the study, we sprayed every 10 min the bone samples with a lukewarm normal saline solution. Next, we made a 3 mm diameter × 11 mm deep pilot drill hole at 800 rpm (Figure 2A). We enlarged it with a second 3.8 mm drill (Figure 2B) under constant water irrigation to control unwanted nearby bone heating, following published recommendations [46]. Finally, we screwed the implants at 15 rpm and 40 Ncm torque (Figure 2C), as advised in [47]. The implant’s superior surface had a mark to orient the barrette’s holes in a perpendicular direction to the calf rib bone (Figure 2D). (Figure 2B). The barrettes were deployed, screwing the central screw (Figure 2E). The idea is that the barrette’s end rests on the lateral cortical bone surfaces (Figure 2F). In human application, these barrettes must be transversal to the mandibula and maxilla to avoid damaging the roots of the neighboring teeth and to rest on the nearby cortical bone.

**Figure 3:**
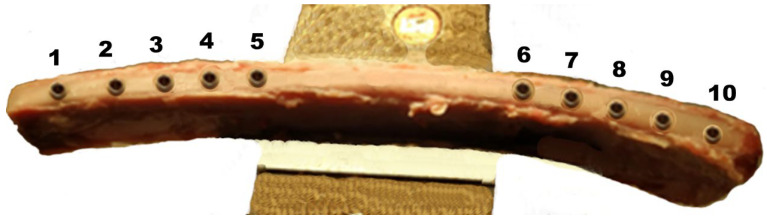
The arrangement of the implants once inserted in a calf rib. Numbering was used to identify each implant during the study.

We inserted ten dental implants into each rib and spaced them 2 cm, as recommended in the literature [29,41,48], keeping the implants’ superior surface flush with the calf rib bone’s surface (Figure 3).

**Figure 4:**
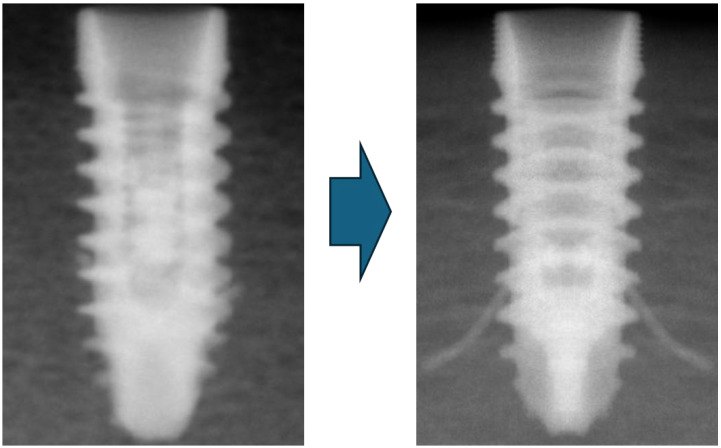
X-ray picture of the dental implant inserted before and after the barrettes were deployed.

We confirmed the correct position of the implant with portable X-ray equipment before deploying the barrettes. In case any implant was not in a perfect position, it was readjusted and confirmed via a new X-ray study before any further steps were taken (Figure 4).

**Figure 5:**
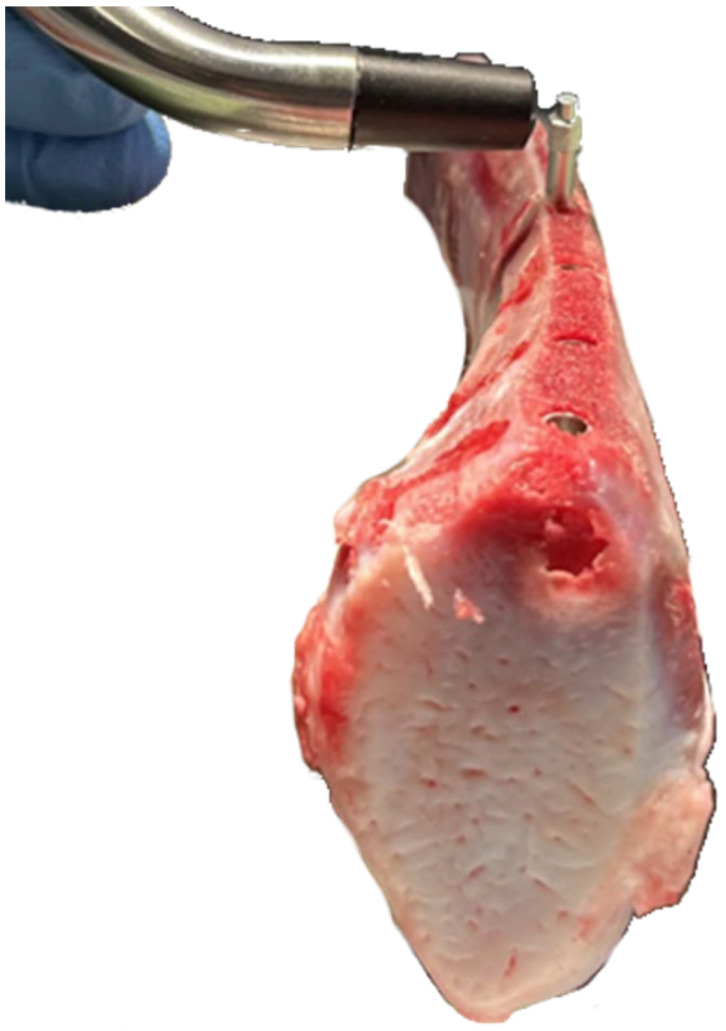
ISQ resonance frequency analysis measurement with Osstell IDx equipment. We screwed the SmartPeg into the crown of our dental implant.

We calculated the Implant Stability Quota (ISQ) with the Osstell IDx resonance frequency analysis (RFA) equipment (Osstell AB, Stampgatan 14, 411 01, Göteborg, Sweden). First, we screwed the SmartPeg (Osstell AB, Stampgatan 14, 411 01, Göteborg, Sweden) in each dental implant and then measured the implants’ frequency vibration in the longitudinal (0°) and transverse (90°) calf rib planes (Figure 5). The ISQ values range between 1 and 100; the higher the ISQ value, the greater the implant stability [49]. In clinical situations, an ISQ value greater than 70 indicates adequate implant stability [50].

We measured the implants’ ISQ before (PRE) and after inserting the barrette (POS) in both transverse and longitudinal calf rib planes.

We introduced the barrettes, screwing their screws with the help of a metric two dynamometric screwdriver with a 5 Ncm maximum torque.

## 3. Statistical Analysis

We used the free statistical analysis software R (R Development Core Team, 2020) in combination with the Deducer user interface [51].

We compared the pairs of measurements and the PRE and POS ISQ values in the calf rib bone transverse and longitudinal planes using the Student’s t-test for related samples (paired *t*-test). For each of these analyses, a new variable was generated, which is the difference between the pairs of measurements. We evaluated whether there were statistically significant differences from the mean difference. Next, we found out if the distribution of our data was normal with the Shapiro–Wilk and the Wilcoxon signed-rank tests. Box and flying buttress diagrams for repeated measures *t*-tests were created, representing the difference of the means (POS-PRE) for each measure. So, the more significant this difference from a specific value (minimum detectable difference) away from zero, the greater the statistical significance. These diagrams allowed us to compare sample shape, core tendency, and variability easily. We also used them to observe the dispersion of data and identify possible outliers.

Statistically, we considered significant differences if *p* < 0.05.

## 4. Results

On each dental implant, we repeated trice the ISQ measurement. Table 1 shows four columns. The first corresponds to the grouped variance. The second is the difference in means that were found. The third column is the size of the effect (which is the same in all cases since it only depends on the design of the experiment). From these two values, the minimum detectable difference is shown in the fourth column.

### 4.1. Calf Rib’s Longitudinal Plane

As mentioned above, for each implant, three repetitions of the measurement of the ISQ value in the calf rib’s longitudinal plane were performed. Table 2 shows the average ISQ value in this plane in the PRE (before barrette deployment) and POS (post-barrette deployment) conditions.

**Figure 6:**
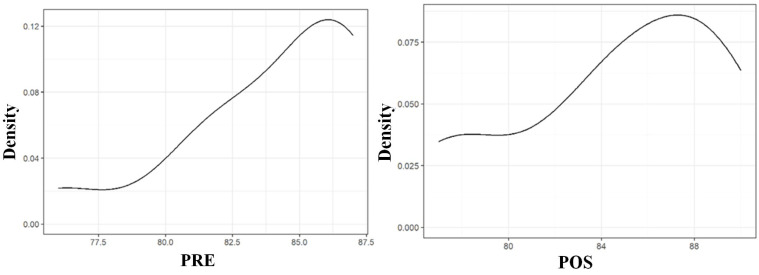
Results of the Shapiro–Wilk normality test of the ISQ data in the calf rib’s longitudinal plane. The ISQ distribution was not normal for the condition PRE (*p* = 0.04), but it was so for the POS (*p* = 0.31).

The Shapiro–Wilk test showed that the ISQ distribution was not normal for the condition PRE (*p* 0.04), but it was so for the POS (*p* 0.31) (Table 3 and Figure 6).

**Figure 7:**
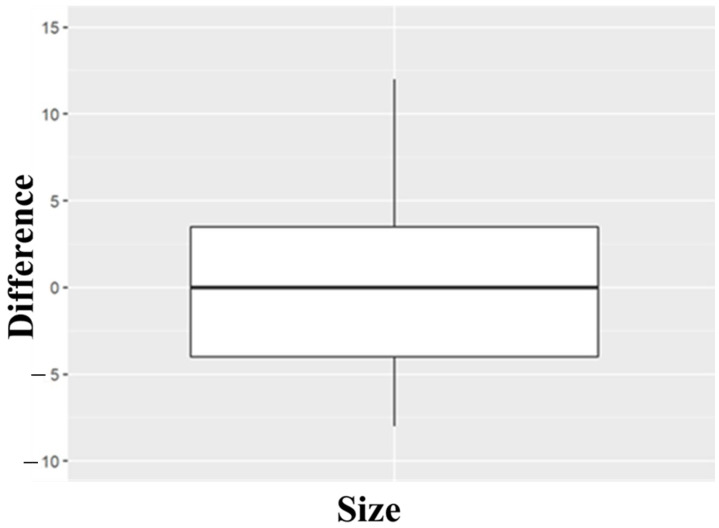
Box plot of ISQ value differences in PRE and POS conditions in the calf rib’s longitudinal plane.

As the distribution of data of the PRE condition was not normal, we alternately used the Wilcoxon test to compare the PRE and POS conditions. The resulting *p*-value was 0.84, higher than the established significance value of 0.05, so no statistically significant differences were found between the two groups. This fact was predictable by looking qualitatively at the table of descriptions (Table 1) given that the difference between ISQ means is only 0.73 over values of around 84 and the difference between the medians is 0. Graphically, it can be seen in the following figure (Figure 7, box plot of the ISQ value difference of PRE and POS conditions in the longitudinal calf rib), where the center line of the box is at a value of 0 since the value of the median is the same in both conditions (85.5).

To summarize the results in the calf rib’s longitudinal plane, the mean ISQ value was PRE 84.00 ± 3.56 and POS 94.73 ± 4.53. The Shapiro–Wilk test showed that the distribution was not normal for the PRE condition (W 0.84, *p* = 0.004) but was normal for the POS (W 0.91, *p* = 0.31). The Wilcoxon signed-rank test with continuity corrections and the resulting *p* 0.84 value confirmed that there were no statistically significant differences between the two groups. This fact was predictable given that the difference between means was only 0.73 on ISQ values around 80, and the difference between medians was 0. Thus, the deployment of the barrettes increases our dental implant primary stability by 2% in the calf rib’s longitudinal plane. This result is logical as the barrette deploys only in the transverse plane and not in the longitudinal one.

### 4.2. Calf Rib’s Transverse Plane

Three repetitions of the measurement of the ISQ value in the transverse plane were also performed for each implant. Table 4 shows the average ISQ value in this plane in the PRE (before barrette deployment) and POS (post-barrette deployment) conditions.

The descriptions of the ISQ value in the calf rib’s transverse plane of the PRE and POS conditions are shown in Table 4.

**Figure 8:**
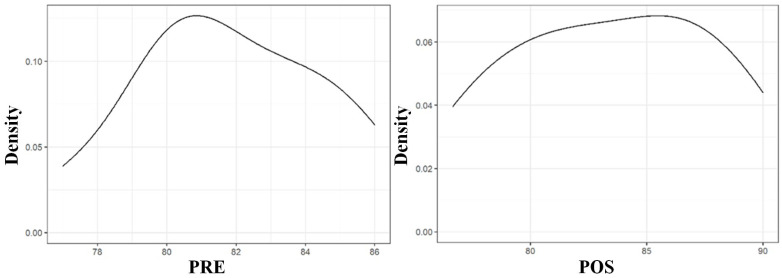
Results of the Shapiro–Wilk normality test of the ISQ data of the transverse plane.

The Shapiro–Wilk test confirmed that the distribution of the data of the PRE and POS conditions was normal (*p*-value above 0.05 in both cases) (Table 5 and Figure 8).

**Figure 9:**
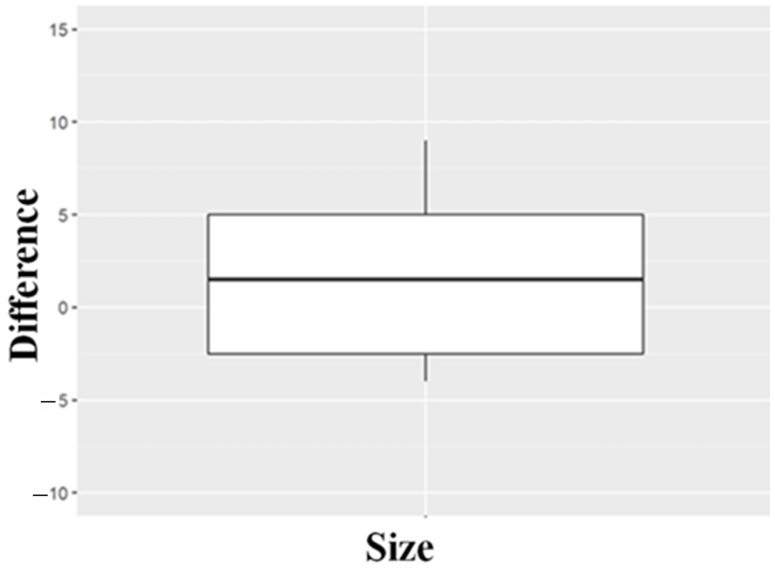
Box plot of the difference of the ISQ value of the PRE and POS conditions in the calf rib’s transverse plane.

Once it was confirmed that the distribution of the PRE and POS condition data was normal, a *t*-test was carried out (Table 6). The resulting *p*-value was 0.27, higher than the established significance value of 0.05. The value resulting from the difference in the means was 11%. This can be seen graphically in the box diagram in Figure 9, which shows the box plot of the difference in the ISQ value of the PRE and POS conditions in the calf rib’s transverse plane.

To summarize the results in the calf rib’s transverse plane, the mean ISQ values were PRE 81.80 ± 2.74 and POS 83.53 ± 4.53. The Shapiro–Wilk test (PRE W 0.96, *p* = 0.83 and POS W 0.96, *p* = 0.77) showed that the distribution was normal for both conditions. The related sample T-test between the PRE and POS ISQ conditions had a t of 1.17, df of 9.00, *p* = 0.27, and mean of differences of 1.73 (95% confidence interval: −1.62 to 5.08). As the resulting *p*-value was 0.27, there were no statistically significant differences between the two groups. The insertion of the barrettes increases our dental implant primary stability in the calf rib’s transverse plane by 11%.

## 5. Discussion

It is known that micromotion facilitates fibrous tissue formation and dental implant failure [27]. In osteoporotic patients, tooth decay is common, and dental implant failure is frequent due to poor-quality bone that has poor osseointegration capacities [7,52]. So, any improvement in primary implant stability is to be welcomed while awaiting secondary and definitive implant osseointegration [53,54]. In this respect, our dental implant design and materials aimed to achieve the strongest possible primary stability that would foster long-term osseointegration [55].

In the present study, the mean value of the ISQ in the calf rib before deploying the barrettes was 84.00 in the longitudinal and 81.80 in the transverse planes. These values are above those reported in studies examining other dental implants [29,40,41,48]. Additionally, barrette deployment increased the primary dental implant stability by 10.75% in the transverse and 2% in the longitudinal calf rib planes. This improvement in primary implant stability might prove particularly helpful to patients with grade IV osteoporotic bone, which is prevalent in older persons [56]. Our first task was to decide how to evaluate the primary dental stability using a non-destructive method such as a pull-out force [57]. We decided to carry this out through the evaluation of the ISQ value via resonance frequency analysis (RFA) because it is commonly used by most research groups nowadays [29,40,48]. This technique is non-invasive, user-friendly, and can be repeated as many times as needed without causing any damage or creating instability in the tested dental implant [58].

The second issue was to find out which primary dental stability measure via ISQ should be expected from a dental implant. The published human values vary between research groups (63.81 ± 9.48 [59], 67.70 ± 5.51 [58], 68.61 ± 10.35 [60], 71.2 ± 10.6 [61], 73.98 ± 5.40 [62], and 75.29 ± 8.76 [23]). There are also differences in lab models. For example, for the dental implant in the femoral head model, it was 63.93 ± 1.10 [63], and in the calf rib, it oscillated between 71 ± 5.49 and 79.1 in the transverse and 73.63 ± 4.88 and 74.5 in the longitudinal planes [40,48]. These differences might be related to the fact that bone quality is not the same in all cases. With these data in hand, we can say that the calf rib dental implant model has an ISQ similar to that expected in humans. These values are below the ones found with our dental implant even before the barrettes’ deployment.

The shape of the dental implant matters with respect to primary stability [64]. Although tapered-shaped ones have the highest ISQ values (75.37 ± 2.06) [8,29], they are lower than those achieved with our implant, even before the barrettes’ deployment.

Another way to increase primary dental implant stability is by an expandable [61] or barbed [57] design. The mean ISQ values of the expandable ones are 71.2 ± 10.6 [61], and for the barbed ones, they are 66.1 ± 8.0 for the maxilla and 75.9 ± 10.6 for the mandible [57]. In both cases, these ISQ values are below the 84 values provided by our dental implant even before barrettes’ deployment. Additionally, the body of our dental implant is porous to facilitate bone ingrowth during the process of osseointegration.

Transverse forces are the most damaging to the bone–implant interface and can lead to the fracture of its components and peri-implant bone resorption [65]. The extra 10% ISQ improvement in the transverse plane provided by the deployment of the two barrettes of our dental implant might provide additional help.

Recipient bone microfracture is a possibility when inserting the dental implant [66], particularly considering the frailty of osteoporotic bone in old edentulous patients [67]. This problem can be circumvented with a careful technique, avoiding any unnecessary stresses during implant insertion [68] and using dental implant threads measuring 0.40 mm in width and 0.05 mm in thickness. Our dental implant’s design took into account this issue [39].

The fatigue failure of dental implants and the fracture of structures in specific areas depend on the type of implant and the design and characteristics of the abutment–implant connections [69]. The conical connection of dental implants, apart from significantly reducing the loosening of the screws, ensures that the forces are transmitted to a larger surface, minimizing biomechanical complications [70].

In dental implants, occlusal overload beyond the physiological limits of the bone can result in deformation, leading to bone resorption through a remodeling process [71]. In this sense, in the bone–implant system, both materials have very different moduli of elasticity, which is why most of the stress transferred to the peri-implant bone is located in the most coronal portion of the bone surrounding the implant neck [72,73]. We took this fact into account when designing our dental implant.

A final consideration is the possibility of medium–long-term barrette fractures with a loss of primary functions, namely, to increase the primary stability of the dental implant. In our implant, the barrettes are made of titanium alloy (Ti6Al4V, elasticity module 1100 MPa), and they are 1.5 mm in diameter. They deploy out of the main body of the dental implant by 4 mm, each in opposite directions [39]. Their ultimate tensile strength is 1170 MPa [74]; the tensile strength yields 1100 MPa [75], elongation at break is 10%, and the modulus of elasticity is 110 GPa [76]. Thus, it is unlikely that the barrettes fracture over the six weeks required for osseointegration. Due to the stiffness of titanium, the most likely possibility is that they bend rather than fracture in the direction of the loads. However, clinical studies are needed to answer this question.

## 6. Limitations

Among them, it has to be stressed that the number of samples was reduced; the tests were performed on animal bones and not human specimens; the bones used for our study were healthy, and they came from young animals and, thus, were not osteoporotic.

Additionally, the tested dental implant is a prototype and not a final product. The instruments used to implant the samples are not explicitly designed for this endeavor.

Finally, we have no data on the long-term results; that is, we have no data on whether the daily dental implant load on the masticatory process will induce metal fatigue with barrette bending or fracture. In this event, we do not know to what extent the potential long-term barrette failure may affect dental implant stability and potential osseointegration. It is quite likely that once osseointegration occurs, the barrette’s anchoring system will no longer be needed. We plan to carry out prospective human clinical studies of osteoporotic mandibular and maxillary bones under clinical loading conditions.

## 7. Strengths

We have compared the PRE and POS conditions to see to what extent the barrette improves the primary stability force of our dental implant.

## 8. Conclusions

Our dental implant ISQ values are in the higher range reported in the literature and reflect high primary stability after insertion. The barrette’s insertion increases our dental implant primary stability by 11% in the transverse plane and 2% in the longitudinal plane.

## Figures and Tables

**Table 1:** Summary of ISQ data in the calf rib’s longitudinal and transverse planes.

ISQ DATA	Grouped Variance	Mean Difference	Effect Size	Minimum Differences
Longitudinal plane	6.19	0.73	1	6.17
Transverse plane	4.68	1.73	1	4.67

**Table 2:** Average ISQ values in the calf rib’s longitudinal plane comparing the conditions PRE and post-barrette POS.

ISQ Mean Values in the Longitudinal Calf Rib Plane		
	PRE	POS
Mean	84.00	84.73
SD	3.56	4.53
Median	85.50	85.50

**Table 3:** Results of the Shapiro–Wilk normality test of the ISQ data in the calf rib’s longitudinal plane.

Shapiro–Wilk Normality Test ISQ Data Distribution in the Calf Rib’s Longitudinal Plane		
	W	*p*-Value
PRE	0.84	0.04
POS	0.91	0.31

**Table 4:** Average ISQ values in the calf rib’s transverse plane comparing the conditions PRE and POS.

ISQ Mean Values in the Calf Rib’s Transverse Plane		
	PRE	POS
Mean	81.00	83.53
SD	2.74	4.53
Median	81.50	83.50

**Table 5:** Results of the Shapiro–Wilk normality test of the ISQ data of the PRE and POS conditions in the calf rib’s transverse plane.

Shapiro–Wilk Normality Test ISQ Data Distribution in the Transverse Calf Rib Plane		
	W	*p*-Value
PRE	0.96	0.83
POS	0.96	0.77

**Table 6:** Related samples’ *t*-test comparing the PRE and POS conditions in the longitudinal plane.

*t*-Test				
t	df	*p*-Value	Mean of the Differences	95% Confidence Interval
1.17	9.00	0.27	1.73	−1.62

## Data Availability

The data presented in this study are available on request from the corresponding author due to privacy reasons.
